# Psoas muscle index and psoas muscle density as predictors of mortality in patients undergoing hemodialysis

**DOI:** 10.1038/s41598-022-14927-y

**Published:** 2022-06-21

**Authors:** Takahiro Yajima, Maiko Arao, Kumiko Yajima

**Affiliations:** 1 Department of Nephrology, Matsunami General Hospital, Gifu, 501-6062 Japan; 2 Department of Internal Medicine, Matsunami General Hospital, Gifu, 501-6062 Japan

**Keywords:** Medical research, Nephrology

## Abstract

This study aimed to investigate the associations of computed tomography (CT)-measured psoas muscle index (PMI: psoas muscle area normalized by height) and psoas muscle density (PMD: average of bilateral psoas muscle CT values [Hounsfield unit (HU)]) with mortality in patients undergoing hemodialysis. We included 188 hemodialysis patients who underwent abdominal CT. PMI and PMD were measured at the third lumbar vertebral level. We found that PMI and PMD were independently associated with the geriatric nutritional risk index and log C-reactive protein, respectively. The optimal cut-off values of PMI and PMD for men and women were 3.39 cm^2^/m^2^ and 41.6 HU, and 2.13 cm^2^/m^2^ and 37.5 HU, respectively. During follow-up (median 3.5 years), 69 patients died. Lower PMI and lower PMD were independently associated with an increased risk of all-cause mortality [adjusted hazard ratio (aHR) 2.05, 95% confidence interval (CI) 1.14–3.68; aHR 3.67, 95% CI 2.04–6.60), respectively]. The aHR for lower PMI and lower PMD vs. higher PMI and higher PMD was 5.34 (95% CI 2.38–11.97). The addition of PMI and PMD to the risk model significantly improved C-index from 0.775 to 0.893 (p < 0.00001). The combination of PMI and PMD may improve mortality prediction in patients undergoing hemodialysis.

## Introduction

Muscle wasting commonly occurs in patients with end-stage renal disease undergoing hemodialysis and is associated with significant morbidity and mortality^1,2^. It may be caused by protein imbalance secondary to inflammation, increased protein catabolism, and insufficient calorie and protein intakes^2^. Protein energy wasting (PEW), a malnutrition state characterized by loss of muscle and fat mass due to chronic inflammation, is also highly prevalent among patients undergoing hemodialysis and is associated with an increased risk of mortality^3–6^. Thus, muscle wasting may partially explain PEW.

In patients undergoing hemodialysis, muscle function, muscle strength, and physical performance that are core elements for a sarcopenia diagnosis are usually low; therefore, the precise measurement of muscle mass is important^7^. Although dual-energy X-ray absorptiometry and bioelectrical impedance analysis are clinically available to estimate muscle mass; hydration status affects the accuracy of these methods^8,9^. Thus, computed tomography (CT), which is not affected by hydration status, is the gold standard method to estimate muscle mass in patients undergoing hemodialysis^10^. However, the use of CT for evaluating body composition is now limited because of its high cost and the radiation exposure associated with this procedure.

We have previously reported that the CT-measured psoas muscle thickness normalized by height, an easily available surrogate marker of muscle mass volume, may be an indicator of PEW and a promising predictor of mortality in patients undergoing hemodialysis^6^. The importance of evaluating both muscle quantity and quality using CT was recently proposed^11–13^.

Regarding muscle quantity, CT-measured sarcopenic indices are widely used to diagnose sarcopenia or muscle wasting, and to predict mortality in patients with various cancers or chronic liver disease^11^. The former include abdominal skeletal muscle index and psoas muscle index (PMI), in which the area of each muscle is adjusted by the square of the height at the level of the third lumbar vertebra (L3). In patients undergoing hemodialysis, PMI may reflect the whole-body skeletal muscle mass and predict mortality^14,15^.

Regarding muscle quality, the CT attenuation value of the abdominal skeletal or psoas muscles is used as a surrogate marker of myosteatosis, defined as increased fat infiltration in the skeletal muscle^11^. CT-measured psoas muscle density (PMD), which measures the average Hounsfield Unit (HU) attenuation of the bilateral psoas muscles, is a promising predictor of mortality in patients with various cancers, type 2 diabetes mellitus, and trauma^16–19^. The association between muscle quality and mortality has never been investigated in patients undergoing hemodialysis. We hypothesized that myosteatosis could be a novel predictor in this population and that the combination of PMI and PMD, which may reflect muscle quantity and muscle quality, respectively, may improve the accuracy of mortality prediction in this population.

Thus, this study aimed to investigate the associations between CT-measured PMI and PMD and all-cause mortality in patients undergoing hemodialysis. We also examined whether the combination of PMI and PMD could improve prediction of mortality in this population. To the best of our knowledge, this is the first study to evaluate the relationship between the combination of PMI and PMD and mortality in patients undergoing hemodialysis.

## Results

### Baseline characteristics

In this retrospective study, we screened 207 patients who had undergone hemodialysis for > 6 months and abdominal CT between January 2008 and December 2017 at the outpatient clinic of Matsunami General Hospital. Abdominal CT was performed as a screening test to detect early-stage renal cell carcinoma within 1 year of initiation of hemodialysis or transfer to our hospital. After excluding 19 patients with a history of cancer, 188 patients undergoing hemodialysis were included. The baseline characteristics of the study participants are summarized in Table 1. The psoas muscle area (PMA) and PMI in men were significantly higher than those in women (11.4 ± 4.5 cm^2^ vs. 6.6 ± 2.7 cm^2^ and 4.2 ± 1.6 cm^2^/m^2^ vs. 2.8 ± 1.1 cm^2^/m^2^, respectively; both p < 0.0001). In contrast, the PMD values were comparable between men and women (42.7 ± 10.1 HU and 42.5 ± 9.4 HU, p = 0.92).

**Table 1:** Baseline characteristics of the study participants (N = 188).

Characteristics	Total (n = 188)	G1 (n = 103)	G2 (n = 20)	G3 (n = 25)	G4 (n = 40)	*p*-value
Age (years)	63.3 ± 13.6	58.4 ± 14.1	68.0 ± 12.2	66.7 ± 8.6	71.5 ± 10.0	< 0.0001
Male (%)	65.4	62.1	50.5	88.0	67.5	0.040
**Underlying kidney disease**						0.46
Diabetic kidney disease (%)	40.4	39.8	30.0	44.0	45.0	
Chronic glomerulonephritis (%)	32.4	35.0	35.0	16.0	35.0	
Nephrosclerosis (%)	19.7	17.5	25.0	36.0	12.5	
Others (%)	7.4	7.8	10.0	4.0	7.5	
Hemodialysis vintage (years)	2.3 (1.0–5.3)	2.2 (0.9–5.1)	3.0 (0.9–5.7)	3.8 (1.6–6.0)	1.5 (0.8–4.8)	0.12
Alcohol (%)	20.7	18.4	15.0	36.0	20.0	0.23
Smoking (%)	23.4	25.2	20.0	28.0	17.5	0.71
Hypertension (%)	95.2	97.1	90.0	96.0	92.5	0.45
Diabetes (%)	43.6	43.7	40.0	44.0	45.0	0.98
History of CVD (%)	64.9	55.3	70.0	80.0	77.5	0.022
Dry weight (kg)	56.6 ± 12.7	58.8 ± 13.2	47.1 ± 8.7	63.5 ± 9.3	51.4 ± 10.5	< 0.0001
Height (cm)	160.2 ± 8.7	160.8 ± 9.0	157.4 ± 7.6	162.0 ± 7.7	158.6 ± 8.6	0.16
Body mass index (kg/m^2^)	21.9 ± 4.0	22.6 ± 4.1	19.0 ± 3.1	24.2 ± 3.0	20.4 ± 3.3	< 0.0001
Blood urea nitrogen (mg/dL)	57.4 ± 14.8	59.0 ± 12.2	62.3 ± 16.0	56.6 ± 15.8	51.2 ± 18.0	0.013
Creatinine (mg/dL)	9.4 ± 3.1	10.2 ± 3.1	9.1 ± 2.4	9.9 ± 2.6	7.4 ± 2.7	< 0.0001
Albumin (g/dL)	3.7 ± 0.5	3.8 ± 0.4	3.7 ± 0.4	3.7 ± 0.3	3.3 ± 0.6	< 0.0001
Hemoglobin (g/dL)	10.6 ± 1.5	10.6 ± 1.5	10.4 ± 1.2	10.7 ± 1.2	10.6 ± 1.7	0.95
Total cholesterol (mg/dL)	151 ± 33	152 ± 32	158 ± 46	158 ± 30	140 ± 29	0.095
Uric acid (mg/dL)	6.9 ± 1.7	7.0 ± 1.6	7.4 ± 1.4	7.1 ± 1.5	6.4 ± 2.1	0.086
Calcium (mg/dL)	9.0 ± 0.8	9.0 ± 0.8	8.8 ± 0.7	9.2 ± 0.9	9.1 ± 1.1	0.37
Phosphorus (mg/dL)	4.9 ± 1.4	5.1 ± 1.3	5.2 ± 1.3	4.8 ± 1.3	4.5 ± 1.6	0.071
iPTH (pg/mL)	113 (48–186)	138 (63–219)	133 (53–243)	83 (49–145)	71 (26–131)	0.0076
Glucose (mg/dL)	142 ± 58	139 ± 58	143 ± 55	151 ± 54	143 ± 63	0.83
C-reactive protein (mg/dL)	0.17 (0.08–0.54)	0.14 (0.06–0.29)	0.12 (0.09–0.12)	0.22 (0.12–0.61)	0.54 (0.15–2.44)	0.0002
Kt/V for urea	1.38 ± 0.29	1.38 ± 0.28	1.56 ± 0.29	1.20 ± 0.21	1.38 ± 0.29	0.0004
GNRI	93.8 ± 8.3	96.5 ± 6.5	91.0 ± 8.5	96.1 ± 5.5	86.8 ± 9.5	< 0.0001
SCI	20.5 ± 3.2	21.4 ± 3.2	19.8 ± 2.3	21.0 ± 2.4	18.4 ± 2.9	< 0.0001
Psoas muscle area (cm^2^)	9.8 ± 4.6	12.0 ± 4.4	5.5 ± 1.8	10.9 ± 2.6	5.5 ± 1.6	< 0.0001
PMI (cm^2^/m^2^)	3.7 ± 1.6	4.5 ± 1.4	2.2 ± 0.6	4.2 ± 0.9	2.2 ± 0.5	< 0.0001
PMD (HU)	42.6 ± 9.9	48.7 ± 4.9	47.0 ± 4.8	33.2 ± 8.5	30.5 ± 5.7	< 0.0001

*PMI* psoas muscle index, *PMD* psoas muscle density, *CVD* cardiovascular disease, *iPTH* intact parathyroid hormone, *GNRI* geriatric nutritional risk index, *SCI* simplified creatinine index, *G1* higher PMI and higher PMD, *G2* lower PMI and higher PMD, *G3* higher PMI and lower PMD, *G4* lower PMI and lower PMD.

### Agreements for the PMA and PMD measurements

The intra- and inter-operator reproducibility of CT-measured PMA and PMD were evaluated in a randomly selected sample of 30 patients; the measurements were blindly performed by two investigators (T.Y. and M.A.). The intra-observer intraclass correlation coefficients (ICCs) for the PMA measurements were 0.99 [95% confidence interval (CI) 0.99–0.99] for both T.Y. and M.A, whereas those for the PMD measurements were 0.97 (95% CI 0.94–0.99) for T.Y. and 0.98 (95% CI 0.96–0.99) for M.A. The inter-observer ICCs for the PMA and PMD measurements were 0.86 (95% CI 0.65–0.95) and 0.86 (95% CI 0.64–0.94), respectively.

### Associations of PMI and PMD with baseline variables

PMD was significantly correlated with PMI (r = 0.445, p < 0.0001). In the univariate regression analysis, both PMI and PMD were significantly and negatively associated with age and log C-reactive protein (CRP), and were significantly and positively associated with geriatric nutritional risk index (GNRI) and SCI. In addition, PMI was also significantly and negatively associated with male sex. In the multivariate regression analysis, PMI was independently associated with male sex and GNRI, whereas PMD was independently associated with age and log CRP (Table 2).

**Table 2:** Regression analysis of the associations of the psoas muscle index and psoas muscle density with baseline variables.

Variables	Psoas muscle index				Psoas muscle density			
	Univariate analysis		Multivariate analysis		Univariate analysis		Multivariate analysis	
	r	*p*-value	β	*p*-value	r	*p-*value	β	*p-*value
Age	− 0.367	< 0.0001	− 0.142	0.094	− 0.450	< 0.0001	− 0.289	0.0001
Male	0.418	< 0.0001	0.309	< 0.0001	NA	NA	NA	NA
GNRI	0.481	< 0.0001	0.322	< 0.0001	0.333	< 0.0001	0.052	0.52
SCI	0.484	< 0.0001	0.113	0.23	0.375	< 0.0001	0.083	0.35
Log CRP	− 0.217	0.0028	− 0.014	0.83	− 0.392	< 0.0001	− 0.244	0.0011

*GNRI* geriatric nutritional risk index, *SCI* simplified creatinine index, *CRP* C-reactive protein, *NA* not applicable.

### Associations of the PMI and PMD with all-cause mortality

**Figure 1:**
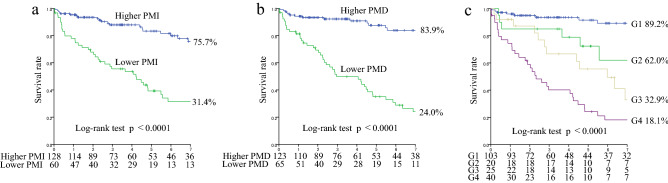
Kaplan–Meier survival curves for all-cause mortality. All-cause mortality for lower PMI vs. higher PMI (**a**), lower PMD vs. higher PMD (**b**), and in the four groups (G1 to G4) with PMI and PMD (**c**). *G1* higher PMI and higher PMD, *G2* lower PMI and higher PMD, *G3* higher PMI and lower PMD, *G4* lower PMI and lower PMD, *PMI* psoas muscle index, *PMD* psoas muscle density.

During the median follow-up of 3.5 [interquartile range (IQR) 1.5–7.1] years, 69 patients died due to cardiovascular disease (CVD) (n = 37, 53.6%), infection (n = 17, 24.6%), cancer (n = 4, 5.8%), and other causes (n = 11, 15.9%). In the univariate Cox proportional hazards analysis, PMI and PMD were identified as significant predictors for all-cause mortality and were significantly associated with all-cause mortality in each sex group [women: Hazard ratio (HR) 0.44, 95% CI 0.25–0.72, p = 0.0007; HR 0.87, 95% CI 0.82–0.91, p < 0.0001; men: HR 0.45, 95% CI 0.34–0.59, p < 0.0001; HR 0.94, 95% CI 0.93–0.97, p < 0.0001, respectively]. To maximize the predictive value of PMI and PMD for all-cause mortality in each sex group, an ROC analysis was performed, which revealed cut-off values of 2.13 cm^2^/m^2^ (sensitivity 0.791; specificity 0.637; AUC 0.742, p = 0.0029) and 37.5 HU (sensitivity 0.930; specificity 0.591; AUC 0.817; p < 0.0001) in women, and 3.39 cm^2^/m^2^ (sensitivity 0.921; specificity 0.660; AUC 0.865; p < 0.0001) and 41.6 HU (sensitivity 0.842; specificity 0.788; AUC 0.840; p < 0.0001) in men, respectively. The 7-year survival rates were 75.7%, 31.4%, 83.9%, and 24.0%, in the higher PMI, lower PMI, higher PMD, and lower PMD groups, respectively (all p < 0.0001) (Fig. 1a,b). In the multivariate Cox proportional hazards analysis adjusted by sex and age, history of CVD, GNRI, SCI, and log CRP, which were significant factors on univariate analysis, lower PMI and lower PMD were independently associated with an increased risk of all-cause mortality, respectively [adjusted HR (aHR) 2.05, 95% CI 1.14–3.68, p = 0.016; aHR 3.67, 95% CI 2.04–6.60, p < 0.0001] (Table 3). The 7-year survival rates were 89.2%, 62.0%, 32.9%, and 18.1%, in G1, G2, G3, and G4, respectively (p < 0.0001) (Fig. 1c). In the multivariate Cox proportional hazards analysis, the aHRs for all-cause mortality were as follows: 1.83 (95% CI 0.67–4.96, p = 0.24) for G2 vs. G1, 3.95 (95% CI 1.60–9.76, p = 0.0029) for G3 vs. G1, and 5.34 (95% CI 2.38–11.97, p < 0.0001) for G4 vs. G1, respectively (Table 3).

**Table 3:** Cox proportional hazards analysis of the psoas muscle index and psoas muscle density for all-cause mortality.

Variables	Univariate analysis		Multivariate analysis^a^	
	HR (95% CI)	*p*-value	HR (95% CI)	*p-*value
**All-cause mortality**				
PMI (continuous)	0.53 (0.43–0.66)	< 0.0001	0.67 (0.52–0.85)	0.0018
PMD (continuous)	0.93 (0.92–0.95)	< 0.0001	0.96 (0.93–0.98)	0.0002
Lower PMI	5.03 (3.05–8.29)	< 0.0001	2.05 (1.14–3.68)	0.016
Lower PMD	7.00 (4.10–11.93)	< 0.0001	3.67 (2.04–6.60)	< 0.0001
Cross-classified (vs. G1)		< 0.0001		< 0.0001
G2	4.81 (1.94–11.88)	0.0007	1.83 (0.67–4.96)	0.24
G3	7.26 (3.20–16.45)	< 0.0001	3.95 (1.60–9.76)	0.0029
G4	14.38 (7.09–29.14)	< 0.0001	5.34 (2.38–11.97)	< 0.0001

*HR* hazard ratio, *PMI* psoas muscle index, *PMD* psoas muscle density, *G1* higher PMI and higher PMD, *G2* lower PMI and higher PMD, *G3* higher PMI and lower PMD, *G4* lower PMI and lower PMD.

^a^Adjusted by gender and age, history of cardiovascular disease, geriatric nutritional risk index, simplified creatinine index, and log of C-reactive protein level.

### Model discrimination

The C-index of all-cause mortality significantly improved in the ascending order of adding PMI alone (0.836), PMD alone (0.877), and both PMI and PMD (0.893) to the established risk model (0.775), including sex, age, history of CVD, GNRI, SCI, and log CRP (Table 4). Similarly, the net reclassification improvement (NRI) and integrated discrimination improvement (IDI) for all-cause mortality improved upon the addition of PMI and/or PMD to the established risk model (Table 4).

**Table 4:** Predictive accuracy of the psoas muscle index and psoas muscle density for all-cause mortality.

Variables	C-index	*p*-value	NRI	*p*-value	IDI	*p*-value
**All-cause mortality**						
Established risk factors^a^	0.775 (0.705–0.845)		Reference			Reference
+ PMI	0.836 (0.773–0.900)	0.0095	0.922	< 0.00001	0.140	< 0.00001
+ PMD	0.877 (0.826–0.929)	0.0005	1.159	< 0.00001	0.199	< 0.00001
+ PMI and PMD	0.893 (0.843–0.942)	< 0.00001	1.058	< 0.00001	0.255	< 0.00001

*NRI* net reclassification improvement, *IDI* integrated discrimination improvement, *PMI* psoas muscle index, *PMD* psoas muscle density.

^a^Gender and age, history of cardiovascular disease, geriatric nutritional risk index, simplified creatinine index, and log of C-reactive protein level.

## Discussion

We demonstrated that PMI and PMD were independently associated with GNRI and log CRP, respectively. Lower PMI and lower PMD were independently associated with an increased risk of all-cause mortality. Patients with both low PMI and low PMD had the worst all-cause survival rate. In addition, the predictability of all-cause mortality improved the most when PMI and PMD were added to the established risk model. Therefore, both PMI and PMD may be surrogate markers of PEW and may be clinically useful to stratify the risk of all-cause mortality and improve the accuracy of mortality prediction in patients undergoing hemodialysis. Thus, our findings suggest the merit of simultaneously measuring these indicators in this population.

Recently, both muscle quantity and quality have attracted attention as important indicators of sarcopenia^11–13^; decreased muscle quality or myosteatosis may lead to lower muscle strength and physical function in patients with chronic kidney disease and those undergoing hemodialysis^20,21^. Muscle quantity is commonly evaluated by measuring the PMA at the L3 level, whereas muscle quality is evaluated by measuring the average CT attenuation value of psoas muscle^12^.

In this study, PMI and PMD were calculated at the L3 level using a cross-sectional CT image. PMI was positively and independently associated with male sex and GNRI. Previous studies have reported that muscle mass volume differed with sex^6,14,17,18^; in this study, the PMI was significantly higher in men than in women. In patients undergoing hemodialysis, PEW is highly prevalent and is associated with increased risks of morbidity and mortality^3–6^. GNRI, which is easily calculable based on the serum albumin level and BMI, is a useful nutritional indicator to stratify the risk of PEW^22–26^; therefore, our results suggest that PMI may be an indicator of PEW.

Conversely, PMD was independently negatively associated with age and log CRP, an inflammatory marker, in this study. Some previous reports showed that muscle quality may deteriorate with age^27,28^. Furthermore, Raj et al. reported that expression of the inflammatory cytokine IL-6 in the muscle intensified muscle protein catabolism and amino acid release, resulting in acute-phase protein synthesis in patients undergoing hemodialysis^29^. The development of chronic inflammation in chronic kidney disease has been described as the consequence of a multifactorial etiology of interactions that emerge in the uremic circumstances. Chronic inflammation is recognized as a component of the uremic phenotype closely linked to PEW^30^; thus, PMD may be also a surrogate maker of PEW. Interestingly, we noted that PMD was significantly correlated with PMI; therefore, our results demonstrate that muscle quantity and quality might have deteriorated to the same extent in our study cohort. However, although PMI differed with sex, PMD did not. This is an observation that we cannot explain adequately. Although no study has examined PMD in hemodialysis patients, several previous studies reported similar findings^16,17^. Moreover, among the four groups reported in this study, G3 (higher PMI and lower PMD) was almost exclusively comprised of male patients, and was unique in terms of including patients with the longest hemodialysis vintage, the highest habit of alcohol consumption and smoking rates, the highest history of CVD, the largest dry weight and BMI, and the lowest Kt/V for urea. This might be because muscle quantity was maintained in these male patients, but their muscle quality deteriorated.

In this study, a lower PMI was independently associated with an increased risk of all-cause mortality in patients undergoing hemodialysis. Several studies have evaluated the associations between PMI and mortality in Japanese patients undergoing hemodialysis. Kurumisawa et al. reported that PMI measured before cardiovascular surgery was a predictor of survival after surgery^14^. Takata et al. recently reported that PMI was correlated with bioelectrical impedance analysis-measured skeletal muscle mass index, and that lower PMI was associated with increased risks of mortality^15^. Our results were similar to those reported previously. In this study, a lower PMD was independently associated with the risk of all-cause mortality in hemodialysis patients. Several studies have demonstrated that PMD can predict mortality in patients with various types of cancer, type 2 diabetes, and trauma^16–19^. As mentioned above, PMD may reflect inflammation, which may lead to PEW; therefore, the measurement of PMD may be clinically useful as a predictor of mortality in this population.

To the best of our knowledge, this study is the first to investigate the relationship of PMI, PMD, and the two combined with mortality in patients undergoing hemodialysis. Patients with a lower PMI and PMD had the worst all-cause mortality rate. Interestingly, the predictive accuracy of all-cause mortality improved the most when both PMI and PMD were added as factors to the baseline risk model. This may be because PMI and PMD are used to evaluate different aspects of PEW and sarcopenia, as mentioned above; therefore, the combination of these indicators may increase the accuracy of mortality prediction. Thus, when CT is used to evaluate muscle wasting, the simultaneous assessment of PMI and PMD may be recommended to stratify the risk of all-cause mortality and predict mortality in patients undergoing hemodialysis. Indeed, the clinical use of CT for measuring body composition may be limited due to concerns of radiation exposure and its high cost. However, CT can be applied to patients who are not able to perform bioimpedance analysis: i.e., patients implanted with a pacemaker. Moreover, when abdominal CT is performed for other purposes, including cancer screening as in the present study, it might also be useful for detecting muscle wasting or for predicting mortality. Therefore, studies that will examine the clinical utility of CT compared with clinically available bioimpedance analysis may be required in the future.

This study had some limitations. First, this retrospective, single-center study included a small number of patients undergoing hemodialysis. Second, only Japanese patients undergoing hemodialysis, who reportedly have a better prognosis than those in the United States of America and Europe^31^, were enrolled, thereby limiting the generalizability of our results to patients undergoing hemodialysis in other countries. Third, the PMI and PMD values used for the data analyses were measured only at enrollment, and any changes in these values during the follow-up period were not evaluated. More considerations are required to decide the optimal cutoff value of PMI and PMD in patients undergoing hemodialysis. Further prospective, large-scale, and multicenter studies are required to validate our results.

In conclusion, CT-measured PMI and PMD were significant predictors for all-cause mortality and may be surrogate markers of PEW in patients undergoing hemodialysis. The combination of PMI and PMD was effective for stratifying the risk of all-cause mortality and improving the accuracy of mortality prediction in this population. Therefore, the simultaneous assessment of these indicators may be recommended to predict mortality in patients undergoing hemodialysis.

## Methods

### Ethical approval

This study was performed in line with the principles of the Declaration of Helsinki and was approved by the ‘Matsunami Generel Hospital Medical Ethics Committee’ (approval number 483). The requirement for informed consent was waived by Matsunami Generel Hospital Medical Ethics Committee owing to the nature of retrospective study design and the analysis of anonymised patient data.

### Data collection

The following data were collected from the patients’ medical records: age, sex, underlying renal disease, duration of hemodialysis, alcohol consumption and smoking history, history of hypertension, diabetes mellitus, and CVD (heart failure, myocardial infarction, stroke, and peripheral artery disease), dry weight, and height. Hypertension was defined as a systolic blood pressure of ≥ 140 mmHg and/or diastolic blood pressure of ≥ 90 mmHg before the hemodialysis sessions or anti-hypertensive drug consumption. Patients with a history of diabetes mellitus or using glucose-lowering medications were considered to have diabetes mellitus. Blood samples were obtained with the patients in supine position before the initiation of a hemodialysis session on a Monday or Tuesday, and the CT and laboratory data collected from the same month were used for data analysis.

### Calculation of nutritional markers^32,33^

The GNRI was calculated as follows: GNRI = 14.89 × serum albumin level (g/dL) + 41.7 × [dry weight (kg)/ideal body weight (kg)]^22^ The ideal body weight (kg) was calculated as 22 × [height (m)]^2^. When the dry weight was equal to or exceeded the ideal body weight, the ratio of dry weight to ideal body weight (dry weight/ideal body weight) was set to 1. The simplified creatinine index (SCI) was calculated as follows: SCI (mg/kg/day) = 16.21 + 1.12 × [0 for female; 1 for male] − 0.06 × age (years) − 0.08 × single pool Kt/V for urea + 0.009 × serum creatinine level (μmol/L)^34^.

### Measurement of PMI and PMD

The PMI and PMD were assessed using abdominal CT performed after a hemodialysis session. Using a wide-bore 16 slice multi-detector CT scanner (LightSpeed RT16; GE Healthcare, Waukesha, WI, USA); 5 mm-thick slices were acquired. A cross-sectional CT image at the level of L3 was selected. The picture archiving and communication system, Xtrek View (J-mac System, Inc., Sapporo, Japan) installed in the electronic medical chart, was used to measure PMA and PMD as previously reported^17–19^. The Polygon tool was used to trace the periphery of the bilateral psoas muscles without CT value thresholding. First, the PMA value was obtained by summing the area of right and left psoas muscles. The PMA was normalized by the height squared to obtain the PMI value. The PMD was then measured based on the average CT value of the bilateral psoas muscles, determined by summing the product of the mean right CT value and right PMA and the product of mean left CT value and left PMA, and dividing this value by the PMA.

### Follow-up study

The study endpoint was all-cause mortality. Using a receiver operating characteristic analysis, the cut-off values of PMI and PMD, which maximally predicted all-cause mortality, were obtained for each sex. Subsequently, patients were divided by each sex-specific cut-off value (lower PMI vs. higher PMI groups and lower PMD vs. higher PMD groups). Thereafter, they were divided into four groups according to these cut-off values (G1: higher PMI and higher PMD; G2: lower PMI and higher PMD; G3: higher PMI and lower PMD; G4: lower PMI and lower PMD). Patients were followed up until December 2018.

### Statistical analysis

Normally distributed variables are expressed as the means ± standard deviations, and non-normally distributed variables are expressed as the medians and IQR. To compare the differences among the four groups, a one-way analysis of variance or the Kruskal–Wallis test for continuous variables and the chi-squared test for categorical variables were used. The intra- and inter-observer reproducibilities of the CT-measured PMA and PMD were examined based on the ICCs and 95% CIs.

To evaluate the correlation between PMI and PMD, Pearson’s correlation coefficient was used. A univariate regression analysis was performed to assess the baseline factors associated with the PMI and PMD, respectively. A multivariate regression analysis was performed using all factors that were significant at p < 0.05 in the univariate analysis.

The Kaplan–Meier method was used to estimate survival, which was analyzed using the log-rank test. HRs and 95% CIs for all-cause mortality were assessed using the Cox proportional hazards regression analysis. The multiple regression model included sex and all covariates that were significant at p < 0.05 in the univariate analysis. The proportional hazards assumption was assessed with log-minus-log plot and Schoenfeld residuals, and no apparent violation of the assumption was detected.

To evaluate whether the prediction of mortality could improve after the addition of the PMI and/or PMD to the baseline model with sex and the covariates that were significant at p < 0.05 in the univariate analysis, the C-index, NRI, and IDI were calculated. The C-index was defined as the area under the ROC curve (AUC) between individual predictive probabilities for mortality and the incidence of mortality, and it was compared between the baseline model and the model including the PMI and/or PMD^35^. The NRI is a relative indicator of the number of patients for whom the predicted probabilities for mortality improve, whereas the IDI represents the average improvement in predicted probabilities for mortality after the addition of variables to the baseline model^36^.

Statistical analyses were performed using SPSS version 24 (IBM Corp., Armonk, NY, USA). Statistical significance was set at p < 0.05.

## Supplementary Information

Supplementary Information.

## Data Availability

The dataset analyzed in the present study is available in this published article as a supplementary information files.
